# Crystal structure of 4-methyl-2,6,7-trioxa-1-phosphabi­cyclo­[2.2.2]octa­ne

**DOI:** 10.1107/S2056989016009993

**Published:** 2016-06-24

**Authors:** Musa A. Said, Bayan L. Al Belewi, David L. Hughes

**Affiliations:** a Chemistry Department, Taibah University, PO Box 30002, Code 14177, Al-Madinah Al-Munawarah, Kingdom of Saudi Arabia; b School of Chemistry, University of East Anglia, Norwich, NR4 7TJ, UK

**Keywords:** crystal structure, bicyclic phosphites, 2,6,7-trioxa-1-phosphabi­cyclo­[2.2.2]octa­ne

## Abstract

The title mol­ecule, C_5_H_9_O_3_P, has a bi­cyclo­[2.2.2] structure with the P atom at the prow and the bridge-head C atom, with the bonded methyl group, at the stern. The three six-membered rings in the bi­cyclo­[2.2.2] structure have essentially identical good boat conformations.

## Abstract 2

The title compound has a bi­cyclo­[2.2.2] structure with the P atom at the prow and the bridge-head C atom, with the bonded methyl group, at the stern. The three six-membered rings in the bi­cyclo­[2.2.2] structure have essentially identical good boat conformations.

## Chemical context   

Phospho­rus-based ligands bind strongly to transition metals and these complexes offer a wide range of properties due to the high volume of accessible substituents (Downing & Smith, 2004 ▸; Tolman, 1977 ▸; Joslin *et al.*, 2012 ▸). Complexation experiments with these ligands can yield mono- or bi-nuclear complexes (van den Beuken *et al.*, 1997 ▸). Phospho­rus-based complexes are an important class of compounds in homogeneous catalysis and coordination chemistry (Downing & Smith, 2004 ▸; Kühl, 2005 ▸). In particular, we have noted inter­esting studies comparing the donor ability of bicyclic phosphites and the related acyclic phosphites; the phospho­rus atom in the former shows more positive charge than in the acyclic phosphites and, hence, the donor ability of bicyclic phosphites is lower than that of the related acyclic phosphites (Vande Griend *et al.*, 1977 ▸; Joslin *et al.*, 2012 ▸). The present work is a continuation of an investigation into the synthesis and study of bi- and tri-cyclic, penta- and hexa-coordinated phospho­ranes to form anionic, neutral and zwitterionic compounds (Said *et al.* 1996 ▸; Timosheva, *et al.* 2006 ▸; Kumara Swamy & Satish Kumar, 2006 ▸). In this paper, we report the synthesis, clean isolation and crystal structure of 4-methyl-2,6,7-trioxa-1-phosphabi­cyclo­[2.2.2]octane (Tolman, 1977 ▸; Joslin *et al.*, 2012 ▸).
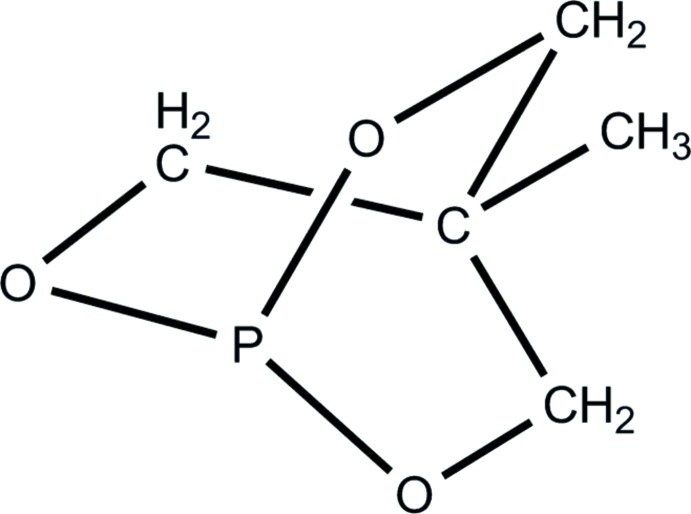


## Structural commentary   

**Figure 1:**
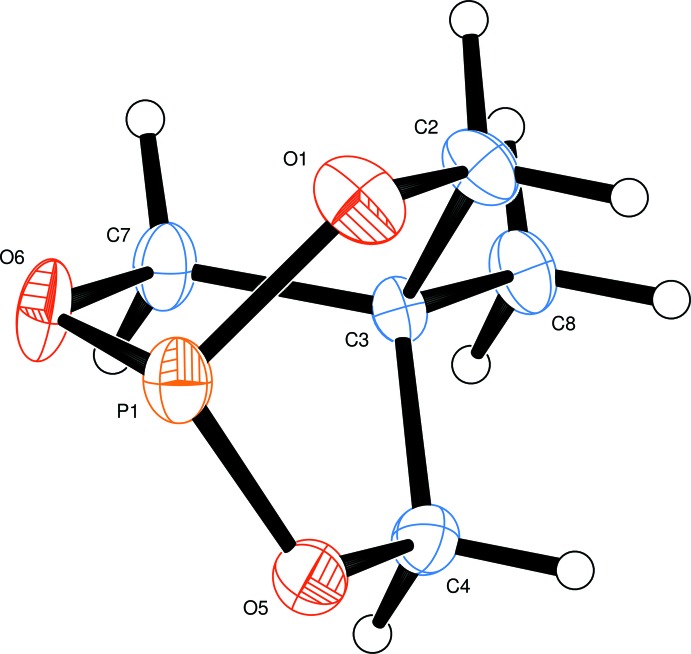
A view of a mol­ecule of *bi­cyclo*-P(OCH_2_)_3_CMe, indicating the atom-numbering scheme. Displacement ellipsoids are drawn at the 30% probability level.

The mol­ecular structure of the title compound, Fig. 1 ▸, shows a bi­cyclo­[2.2.2] structure with the phospho­rus atom as one bridge-head atom and C3, with the bonded methyl group, as the other. The three six-membered rings in the bi­cyclo­[2.2.2] structure have essentially identical, good boat conformations. The P—O bond lengths are very similar, lying in the range 1.613 (2)–1.616 (2) Å; the O—P—O angles at the prow have angles in the range 100.17 (9)–101.34 (10)°, whereas the C—P—C angles at the stern lie in the range 107.99 (17)–109.08 (18)°.
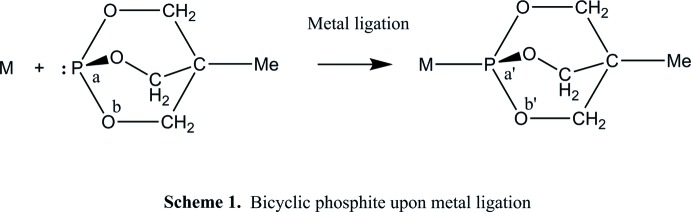


A comparison between acyclic and bicyclic phosphites based on the ‘hinge’ effect has shown (Vande Griend *et al.*, 1977 ▸; Joslin *et al.*, 2012 ▸) that the O—P—O and P—O—C angles, a and b in Scheme 1, change upon ligation with a metal. Due to the steric profile of the bicyclic phosphite, the changes here in a, a′ and b, b′ upon metal ligation are less than in acyclic phosphites. Verkade had pointed out earlier that the *p*-orbital overlap between P and O in bicyclic phosphites is less than in acyclic phosphites, making P more positive and therefore reducing the basicity of P relative to that in acyclic phosphites (Vande Griend *et al.*, 1977 ▸); hence, the coordination ability of acyclic phosphites is higher than that of bicyclic phosphites (Verkade, 1972 ▸). A variety of multi-cyclic phospho­rus compounds including their coordination to various metals has been studied. Based on the trends found in basicity, it is expected that the title compound would show a donating ability to metal centres very similar to that of the more commonly studied bicyclic phosphite P(OCH_2_)_3_CEt (Verkade, 1972 ▸). The average of O—P—O bond angle (a, Scheme 1) in our study is 100.7^o^, whereas the average O—P—O bond angle in coordinated phosphites (a′, Scheme 1) is larger, *e.g.* in Ru{P(OCH_2_)_3_CEt}Cl_2_, it is 102.5^o^ (Joslin *et al.*, 2012 ▸), the same as in [Rh_2_I_2_(C_6_H_5_N_2_O_2_)_2_(COMe)_2_{P(OCH_2_)_3_CMe}_2_] (Venter *et al.*, 2009 ▸); this suggests a slightly larger Tolman angle (Tolman *et al.*, 1977 ▸) after metal ligation. In another study, the enhanced π-accepting ability of the bicyclic phosphite ligand compared to the PPh_3_ and other phosphine ligands was demonstrated clearly in the shorter *M*—P bond distances in the bicyclic phosphite complexes (Erasmus *et al.*, 1998 ▸).

## Supra­molecular features   

**Figure 2:**
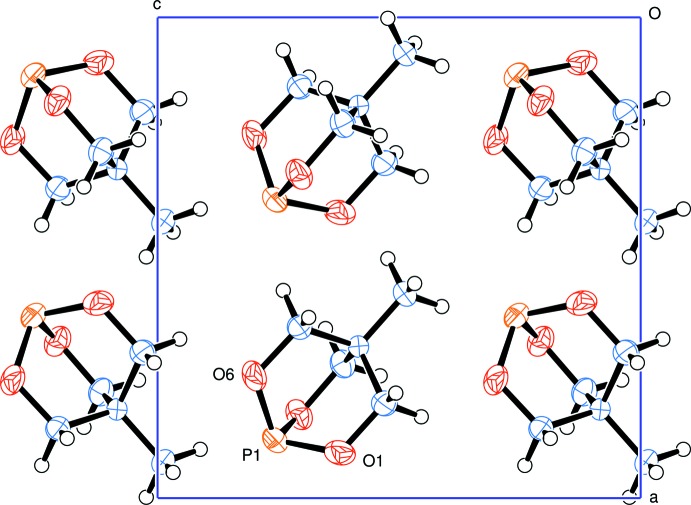
A view along the crystallographic *b* axis.

Contacts between mol­ecules are at normal van der Waals distances, the shortest of which is H4*B*⋯O6′, at 2.58 Å (Table 1 ▸). The nearest neighbours of the phospho­rus atom are hydrogen atoms at distances of at least 3.09 Å. A view of the packing along the *b* axis is shown in Fig. 2 ▸.

## Database survey   

From a selection of crystal structure results for bicyclic phosphites from the Cambridge Structural Database (Groom *et al.*, 2016 ▸), we note that the P—O bond distances:

1) are shortest in phospho­nium ions, as in [Ph_3_C{P(OCH_2_)_3_CMe}]^+^ (Fang *et al.*, 2000 ▸), at *ca* 1.552 Å,

2) in the phosphates, as O=P(OCH_2_)_3_C*R*, (*e.g.* Nimrod *et al.*, 1968 ▸; Santarsiero, 1992 ▸) are *ca* 1.57 Å,

3) in the metal-coordinated phosphites, *M*–{P(OCH_2_)_3_CR} (*e.g.* Aroney *et al.*, 1994 ▸; Venter *et al.*, 2009 ▸; Davis & Verkade, 1990 ▸; Predvoditelev *et al.*, 2009 ▸; Basson *et al.*, 1992 ▸; Erasmus *et al.*, 1998 ▸; Joslin *et al.*, 2012 ▸; Albright *et al.*, 1977 ▸) are *ca* 1.59 Å, and

4) in our results, correlate with those of other unsubstituted phosphites, (*e.g.* Wojczykowski & Jutzi, 2006 ▸; Milbrath *et al.*, 1976 ▸; Predvoditelev *et al.*, 2009 ▸) with P—O bond lengths of *ca* 1.62 Å.

Within each group, there is very little variation in the P—O distances. The bond angles in the bicyclic structure are quite constrained, but we do note a trend, down the four groups of increasing P—O distances, of a corresponding decrease in O—P—O angles from *ca* 107 to 100°.

## Synthesis and crystallization   

To 4.26 g (35.46 mmol) of 2-(hy­droxy­meth­yl)-2-methyl­propane-1,3-diol in 70 mL of dry benzene at RT was added 4.26 g (106.38 mmoles in mineral oil 60%) of NaH in small portions over a period of 20 minutes. The mixture was stirred for 3h before 4.87 g (35.46 mmol) of PCl_3_ were added dropwise over a period of 20 mins in benzene (10 mL) using a dropping funnel. The reaction mixture was stirred overnight before NaCl was removed by filtration under nitro­gen cover. Benzene was removed completely under low pressure. 5 mL of diethyl ether was added, followed by 3 mL of *n*-hexane. The mixture was placed in deep freeze to afford the title compound as a white solid (yield 4.52 g, 86%; m.p. 369–373 K). The product was purified further by sublimation at 393 K/0.5 mm to yield crystals. ^1^H NMR (CDCl3, 400 MHz): 0.73 (*s*, 3H, CH_3_), 3.94 (*s*, 6H, CH_2_). ^13^C NMR (CDCl_3_, 400 MHz): 16.60 (*s*, 1C, CH3), 31.98 [*d*, 1C, C(CH_3_)_3_], 71.80 (*s*, 3C, CH_2_). ^31^P NMR (CDCl_3_, 400 MHz): 91.45 p.p.m. IR cm^−1^: 2950, 1380. Elemental analysis: calculated: C, 40.55; H, 6.13; found: C, 40.83; H, 6.19.

## Refinement   

Crystal data, data collection and structure refinement details are summarized in Table 2 ▸.

The H atoms were included in idealized positions and treated as riding atoms: C—H = 0.93– 0.97 Å with *U*_iso_(H) = 1.5U_eq_(C) for methyl H atoms and = 1.2*U*_eq_(C) for methyl­ene H atoms.

## Supplementary Material

Crystal structure: contains datablock(s) I. DOI: 10.1107/S2056989016009993/lh5816sup1.cif

Structure factors: contains datablock(s) I. DOI: 10.1107/S2056989016009993/lh5816Isup2.hkl

Supporting information file. DOI: 10.1107/S2056989016009993/lh5816Isup3.cdx

Supporting information file. DOI: 10.1107/S2056989016009993/lh5816Isup4.cml

CCDC reference: 1486648

Additional supporting information: crystallographic information; 3D view; checkCIF report

## Figures and Tables

**Table 1:** Hydrogen-bond geometry (Å, °)

*D*—H⋯*A*	*D*—H	H⋯*A*	*D*⋯*A*	*D*—H⋯*A*
C4—H4*B*⋯O6^i^	0.97	2.58	3.495 (4)	158

Symmetry code: (i) 
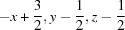
.

**Table 2:** Experimental details

Crystal data	
Chemical formula	C_5_H_9_O_3_P
*M*_r_	148.09
Crystal system, space group	Orthorhombic, *P* *n* *a*2_1_
Temperature (K)	140
*a*, *b*, *c* (Å)	10.4408 (6), 6.2129 (5), 10.5052 (5)
*V* (Å^3^)	681.45 (7)
*Z*	4
Radiation type	Mo *K*α
μ (mm^−1^)	0.34
Crystal size (mm)	0.65 × 0.17 × 0.07

Data collection	
Diffractometer	Oxford Diffraction Xcalibur 3/Sapphire3 CCD
Absorption correction	Multi-scan (*CrysAlis PRO*; Agilent, 2013 ▸)
*T*_min_, *T*_max_	0.684, 1.000
No. of measured, independent and observed [*I* > 2σ(*I*)] reflections	10309, 1561, 1405
*R*_int_	0.043
(sin θ/λ)_max_ (Å^−1^)	0.649

Refinement	
*R*[*F* ^2^ > 2σ(*F* ^2^)], *wR*(*F* ^2^), *S*	0.033, 0.084, 1.11
No. of reflections	1561
No. of parameters	82
No. of restraints	1
H-atom treatment	H-atom parameters constrained
Δρ_max_, Δρ_min_ (e Å^−3^)	0.24, −0.12
Absolute structure	Flack *x* determined using 605 quotients [(*I* ^+^)−(*I* ^−^)]/[(*I* ^+^)+(*I* ^−^)] (Parsons *et al.*, 2013 ▸)
Absolute structure parameter	0.07 (6)

Computer programs: *CrysAlis PRO* (Agilent, 2013 ▸), *SHELXS97* (Sheldrick, 2008 ▸), *SHELXL2014* (Sheldrick, 2015 ▸), *ORTEPIII* (Johnson, 1976 ▸) and *ORTEP-3 for Windows* (Farrugia, 2012 ▸) and *WinGX* (Farrugia, 2012 ▸).

## supplementary crystallographic information

### Crystal data

**Table:**

C_5_H_9_O_3_P	*D*_x_ = 1.443 Mg m^−^^3^
*M**_r_* = 148.09	Mo *K*α radiation, λ = 0.71073 Å
Orthorhombic, *P**n**a*2_1_	Cell parameters from 2685 reflections
*a* = 10.4408 (6) Å	θ = 3.3–32.3°
*b* = 6.2129 (5) Å	µ = 0.34 mm^−^^1^
*c* = 10.5052 (5) Å	*T* = 140 K
*V* = 681.45 (7) Å^3^	Prism, colourless
*Z* = 4	0.65 × 0.17 × 0.07 mm
*F*(000) = 312	

### Data collection

**Table:**

Oxford Diffraction Xcalibur 3/Sapphire3 CCD diffractometer	1561 independent reflections
Radiation source: Enhance (Mo) X-ray Source	1405 reflections with *I* > 2σ(*I*)
Graphite monochromator	*R*_int_ = 0.043
Detector resolution: 16.0050 pixels mm^-1^	θ_max_ = 27.5°, θ_min_ = 3.8°
Thin slice φ and ω scans	*h* = −13→13
Absorption correction: multi-scan (CrysAlis PRO; Agilent, 2013)	*k* = −8→8
*T*_min_ = 0.684, *T*_max_ = 1.000	*l* = −13→13
10309 measured reflections	

### Refinement

**Table:**

Refinement on *F*^2^	Secondary atom site location: difference Fourier map
Least-squares matrix: full	Hydrogen site location: inferred from neighbouring sites
*R*[*F*^2^ > 2σ(*F*^2^)] = 0.033	H-atom parameters constrained
*wR*(*F*^2^) = 0.084	*w* = 1/[σ^2^(*F*_o_^2^) + (0.0459*P*)^2^ + 0.0252*P*] where *P* = (*F*_o_^2^ + 2*F*_c_^2^)/3
*S* = 1.11	(Δ/σ)_max_ < 0.001
1561 reflections	Δρ_max_ = 0.24 e Å^−^^3^
82 parameters	Δρ_min_ = −0.12 e Å^−^^3^
1 restraint	Absolute structure: Flack *x* determined using 605 quotients [(*I*^+^)-(*I*^-^)]/[(*I*^+^)+(*I*^-^)] (Parsons *et al.*, 2013)
Primary atom site location: structure-invariant direct methods	Absolute structure parameter: 0.07 (6)

### Special details

**Table:**

Experimental. CrysAlisPro, Agilent Technologies, Version 1.171.36.21 Empirical absorption correction using spherical harmonics, implemented in SCALE3 ABSPACK scaling algorithm.
Geometry. All esds (except the esd in the dihedral angle between two l.s. planes) are estimated using the full covariance matrix. The cell esds are taken into account individually in the estimation of esds in distances, angles and torsion angles; correlations between esds in cell parameters are only used when they are defined by crystal symmetry. An approximate (isotropic) treatment of cell esds is used for estimating esds involving l.s. planes.

### Fractional atomic coordinates and isotropic or equivalent isotropic displacement parameters (Å^2^)

**Table:**

	*x*	*y*	*z*	*U*_iso_*/*U*_eq_	
P1	0.87893 (7)	0.45449 (13)	0.75670 (8)	0.0380 (2)	
O1	0.9057 (2)	0.5737 (4)	0.6228 (3)	0.0470 (6)	
C2	0.8029 (3)	0.5722 (6)	0.5302 (3)	0.0373 (7)	
H2A	0.8308	0.4974	0.4541	0.045*	
H2B	0.7816	0.7189	0.5068	0.045*	
C3	0.6847 (2)	0.4614 (5)	0.5841 (2)	0.0253 (5)	
C4	0.7208 (3)	0.2300 (4)	0.6164 (3)	0.0349 (6)	
H4A	0.6473	0.1566	0.6524	0.042*	
H4B	0.7456	0.1549	0.5393	0.042*	
O5	0.8263 (2)	0.2254 (4)	0.7070 (2)	0.0399 (5)	
O6	0.7460 (2)	0.5660 (4)	0.7998 (2)	0.0457 (7)	
C7	0.6445 (3)	0.5749 (6)	0.7058 (3)	0.0371 (7)	
H7A	0.6242	0.7240	0.6871	0.045*	
H7B	0.5681	0.5070	0.7397	0.045*	
C8	0.5752 (3)	0.4642 (6)	0.4878 (3)	0.0370 (7)	
H8A	0.6017	0.3921	0.4113	0.055*	
H8B	0.5021	0.3919	0.5231	0.055*	
H8C	0.5530	0.6105	0.4683	0.055*	

### Atomic displacement parameters (Å^2^)

**Table:**

	*U*^11^	*U*^22^	*U*^33^	*U*^12^	*U*^13^	*U*^23^
P1	0.0322 (4)	0.0504 (4)	0.0314 (4)	0.0023 (3)	−0.0076 (4)	−0.0037 (5)
O1	0.0297 (11)	0.0630 (15)	0.0483 (14)	−0.0148 (10)	−0.0050 (10)	0.0112 (12)
C2	0.0313 (15)	0.047 (2)	0.0340 (16)	−0.0046 (13)	−0.0002 (13)	0.0088 (13)
C3	0.0232 (12)	0.0319 (13)	0.0209 (13)	−0.0003 (11)	−0.0002 (10)	−0.0004 (11)
C4	0.0391 (16)	0.0332 (15)	0.0323 (14)	0.0001 (13)	−0.0036 (12)	−0.0026 (13)
O5	0.0448 (12)	0.0399 (12)	0.0350 (11)	0.0094 (10)	−0.0096 (9)	0.0025 (9)
O6	0.0455 (13)	0.0625 (17)	0.0292 (11)	0.0128 (11)	−0.0079 (9)	−0.0209 (10)
C7	0.0295 (16)	0.0508 (18)	0.0310 (14)	0.0082 (13)	−0.0008 (12)	−0.0080 (13)
C8	0.0305 (16)	0.053 (2)	0.0274 (15)	−0.0001 (14)	−0.0049 (12)	0.0014 (13)

### Geometric parameters (Å, º)

**Table:**

P1—O5	1.613 (2)	C4—O5	1.456 (4)
P1—O1	1.614 (3)	C4—H4A	0.9700
P1—O6	1.616 (2)	C4—H4B	0.9700
O1—C2	1.449 (4)	O6—C7	1.450 (4)
C2—C3	1.522 (4)	C7—H7A	0.9700
C2—H2A	0.9700	C7—H7B	0.9700
C2—H2B	0.9700	C8—H8A	0.9600
C3—C7	1.519 (4)	C8—H8B	0.9600
C3—C4	1.524 (4)	C8—H8C	0.9600
C3—C8	1.527 (4)		
			
O5—P1—O1	100.46 (13)	C3—C4—H4A	109.5
O5—P1—O6	100.17 (13)	O5—C4—H4B	109.5
O1—P1—O6	101.34 (14)	C3—C4—H4B	109.5
C2—O1—P1	117.00 (18)	H4A—C4—H4B	108.1
O1—C2—C3	110.7 (2)	C4—O5—P1	116.88 (18)
O1—C2—H2A	109.5	C7—O6—P1	116.95 (18)
C3—C2—H2A	109.5	O6—C7—C3	110.7 (2)
O1—C2—H2B	109.5	O6—C7—H7A	109.5
C3—C2—H2B	109.5	C3—C7—H7A	109.5
H2A—C2—H2B	108.1	O6—C7—H7B	109.5
C7—C3—C2	109.1 (3)	C3—C7—H7B	109.5
C7—C3—C4	108.6 (2)	H7A—C7—H7B	108.1
C2—C3—C4	108.0 (2)	C3—C8—H8A	109.5
C7—C3—C8	110.2 (2)	C3—C8—H8B	109.5
C2—C3—C8	110.8 (2)	H8A—C8—H8B	109.5
C4—C3—C8	110.1 (2)	C3—C8—H8C	109.5
O5—C4—C3	110.5 (2)	H8A—C8—H8C	109.5
O5—C4—H4A	109.5	H8B—C8—H8C	109.5
			
O5—P1—O1—C2	50.4 (3)	C3—C4—O5—P1	1.8 (3)
O6—P1—O1—C2	−52.3 (3)	O1—P1—O5—C4	−52.8 (2)
P1—O1—C2—C3	2.4 (4)	O6—P1—O5—C4	50.8 (2)
O1—C2—C3—C7	57.2 (3)	O5—P1—O6—C7	−54.0 (3)
O1—C2—C3—C4	−60.7 (3)	O1—P1—O6—C7	49.0 (3)
O1—C2—C3—C8	178.7 (3)	P1—O6—C7—C3	3.3 (4)
C7—C3—C4—O5	−60.0 (3)	C2—C3—C7—O6	−60.4 (3)
C2—C3—C4—O5	58.2 (3)	C4—C3—C7—O6	57.1 (3)
C8—C3—C4—O5	179.2 (2)	C8—C3—C7—O6	177.8 (3)

### Hydrogen-bond geometry (Å, º)

**Table:**

*D*—H···*A*	*D*—H	H···*A*	*D*···*A*	*D*—H···*A*
C4—H4*B*···O6^i^	0.97	2.58	3.495 (4)	158

Symmetry code: (i) −*x*+3/2, *y*−1/2, *z*−1/2.
